# Recent trends in emerging strategies for ferroptosis-based cancer therapy

**DOI:** 10.1039/d2na00719c

**Published:** 2023-02-14

**Authors:** Hongli Yu, Jianqin Yan, Zhipeng Li, Limian Yang, Fang Ju, Yong Sun

**Affiliations:** a Department of Pharmaceutics, School of Pharmacy, Qingdao University Qingdao 266073 China sunyong@qdu.edu.cn; b Qingdao Mingde School Qingdao 266000 China; c Department of Oncology, The Second Affiliated Hospital of Qingdao University Qingdao 266000 China jufangjufang@sina.com

## Abstract

Ferroptosis, an iron-dependent mode of regulated cell death, is induced by lipid peroxidation, whose occurrence and execution are primarily controlled by metabolism of iron, lipids, amino acids and glutathione. In recent years, the fast-growing studies of ferroptosis in cancer have promoted its application in cancer therapy. So, this review focuses on the feasibility and characteristics of initiating ferroptosis for cancer therapy, as well as the main mechanism of ferroptosis. And various emerging strategies of cancer therapy based on ferroptosis are then highlighted to describe their design, mechanism of action, and anticancer applications. In addition ferroptosis in diverse cancer types is summarized, some considerations for the research of various preparations that can cause ferroptosis are introduced, and this emerging field is discussed in terms of its challenges and future development directions.

## Abstract 2

Ferroptosis, an iron-dependent mode of regulated cell death, is induced by lipid peroxidation, whose occurrence and execution are primarily controlled by metabolism of iron, lipids, amino acids and glutathione.

## Graphical abstract

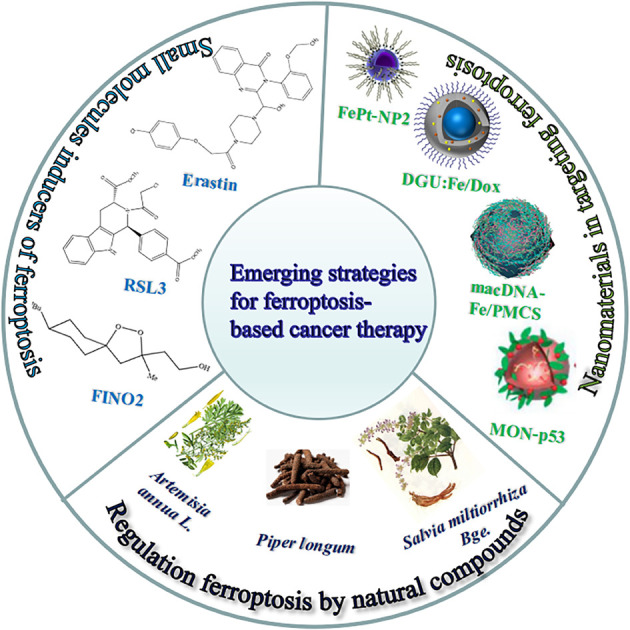

## 1. Introduction

Cell death is mainly divided into two categories: accidental cell death and regulated cell death. The former is the instantaneous catastrophic death of cells after exposure to extreme physical and chemical conditions, such as necrosis; the latter is cell death that relies on the regulation of intracellular molecular mechanisms and can be interfered with by pharmacology and genetics, such as apoptosis.^1^ Scientists gradually discovered the forms of cell death between necrosis and apoptosis, such as necroptosis, pyroptosis, ferroptosis,^2^ autophagic cell death^1^ and so on. Among them, ferroptosis as a new form of regulated cell death has gradually entered our field of vision and has become a research hotspot in the direction of cell death. In the field of tumour therapy, since more and more tumour cells exhibit anti-apoptotic properties,^3^ exploring ferroptosis-related therapeutic methods has potential application value.

**Fig. 1:**
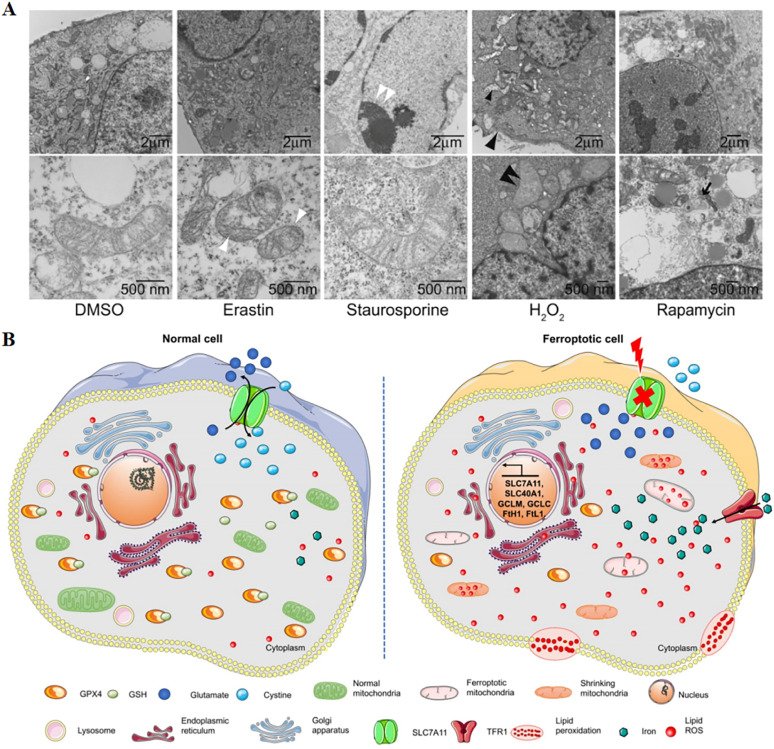
(A) Transmission electron microscopy of BJeLR cells treated with DMSO, erastin (induces ferroptosis), staurosporine (induces apoptosis), hydrogen peroxide (induces necrosis) and rapamycin (induces autophagy). Single white arrowhead, mitochondrial atrophy; a pair of white arrowheads, condensed chromatin; black arrowhead, cytoplasm and organelle swelling and plasma membrane rupture; black double arrowheads, vesicles with a double membrane form. Reproduced with permission.^4^ Copyright 2012, Elsevier Inc. (B) Changes in morphology and biological properties during ferroptosis, mainly including mitochondrial contraction, excess ROS production, iron overload, and intracellular GSH depletion. Reproduced with permission.^7^ Copyright 2020, Ivyspring International Publisher.

A critical role was played by ferroptosis in cancer treatment, which was first proposed by Dixon in 2012,^4^ induced by excess intracellular iron. In ferroptosis, reactive oxygen species (ROS) and lipid hydroperoxides accumulate to lethal levels, causing oxidative damage to cell membranes. The process of ferroptosis differs significantly from that of apoptosis, necroptosis, and autophagy in a number of ways.^5^ Although its mechanism has not been demonstrated, phospholipid molecules containing long chains of unsaturated fatty acids on the cell membrane or organelle membrane are damaged by peroxidation after the inactivation of the intracellular reduction system, resulting in cell membrane rupture, which is considered to be a key feature of ferroptosis. This process can be catalyzed by a Fenton-like reaction with iron as a catalyst.^6^ The most distinct morphological feature between ferroptosis and other types of cell death is the change in mitochondrial morphology (Fig. 1), including reduced or absent mitochondrium cristae, ruptured outer mitochondrial membranes, and dense mitochondrial membranes under an electron microscope.^4,7^ On the other hand, biochemically, the mechanism of ferroptosis is related to the level of intracellular iron metabolism, lipid peroxide content and glutathione peroxidase 4 (GPX4) activity.

Specific changes in cell morphology and biology during ferroptosis are shown in Table 1. By promoting ROS production, p53 acts as a tumour suppressor gene that regulates ferroptosis. As a result of ROS-mediated ferroptosis, p53 directly controls cell metabolism through mitochondrial respiration.^8^ Studies have found that some highly aggressive malignancies are inherently more sensitive to ferroptosis.^9^ Due to its non-apoptotic nature, ferroptosis is expected to overcome the shortcomings of traditional therapies mediated by the apoptotic pathway and has become a new cancer treatment strategy. At present, Wang *et al.* found that CD8+ T cells are activated by immunotherapy enhanced ferroptosis-specific lipid peroxidation in tumour cells, which in turn contributed to the anti-tumour efficacy of immunotherapy.^10^ It was clear from all of this that induced ferroptosis can be beneficial in treating cancer.

**Table 1:** Changes in morphology and biological properties during ferroptosis

	Mitochondrion	GSH	GPX4	ROS	Iron content	TFR	Cystine/glutamate transporter
Normal cells	Normal	Normal	Normal	Normal	Normal	No	Normal
Ferroptotic cells	Reduced mitochondrial cristae, ruptured outer mitochondrial membranes, and dense mitochondrial membranes	Depletion	Reduced	Excess	Overload	Yes	Interrupted

Various strategies based on ferroptosis have been exhibited or developed, including small molecules targeting the ferroptosis pathway, natural compounds, nanomaterials, and targeting the tumour micro-environment. Ferroptosis was originally defined by a group of small molecules called ras-selective lethal compounds (RSLs) that induced selective death of RAS-mutated tumour cells.^11^ At same time, growing evidence suggested that natural compounds, for instance saponins, terpenoids and alkaloids, could induce ferroptosis, further extending their cancer therapeutic potential.^12^ The development of effective nanomaterial carriers is crucial for improving drug delivery, release, and targeting efficiency. In recent years, strategies to deliver drugs targeting ferroptosis using nanomaterials have been extensively studied. Nanomaterials offer new possibilities for triggering ferroptosis in cancer treatment due to their unique physicochemical properties.^13^ In addition, many of the previously reported nanoplatforms, especially those mentioning the generation of ROS by Fenton chemistry, were likely to be related to the iron corrosion mechanism and deserved to be re-evaluated from new perspectives.

In view of the huge potential of ferroptosis in cancer treatment and the rapid development of strategies based on ferroptosis in the last several years, it is of great significance to summarize the latest research work and continuously keep track of the progress in this field. At the same time, the complexity of biological systems and the challenges of clinical transformation have produced challenges and opportunities to make further efforts on ferroptosis-based cancer treatment. This report first summarizes the three main mechanisms of ferroptosis, including iron metabolism, amino acid metabolism and lipid metabolism. It then focuses on various emerging strategies based on ferroptosis (including small molecules, natural compounds, nanomaterials, and the tumour micro-environment) that cause ferroptosis, describing their mechanisms of action and anticancer applications. Subsequently, this paper summarizes ferroptosis in different cancer types. Finally, some thoughts on studying various strategies that can cause ferroptosis are introduced, and this emerging field is discussed in terms of its challenges and future directions.

## 2. Mechanisms of ferroptosis

The regulatory mechanism of ferroptosis involves various signal molecules and metabolic pathways, and is a complex regulatory process. The role of iron, amino acid, and lipid metabolism in ferroptosis pathogenesis is examined in this paper.

### 2.1 Iron metabolism

Iron is one of the essential elements of the human body and the main material to maintain life. Iron participates in the synthesis of hemoglobin, myoglobin, cytochromes, iron–sulfur clusters and many enzymes. Therefore, it plays a crucial role in many fundamental life processes.^14^ Free iron is directly related to ferroptosis as it promotes ROS production through the Fenton reaction leading to lipid peroxidation.^15^ Fe^2+^ is transported out of cells under the action of a membrane iron transporter, and oxidized to Fe^3+^ by copper oxidase. Fe^3+^ combines with transferrin to form a TF–Fe^3+^ complex, which is transported to various tissues and organs through blood circulation.^16^ Meanwhile, Fe^3+^ is transported into the cell after binding with transferrin receptor (TFR) on the cell membrane. The intracellular metal reductase reduces Fe^3+^ to Fe^2+^, and then releases Fe^2+^ into the labile iron pools of the cytoplasm through divalent metal transporter 1 (DMT1). In the normal physiological state, labile iron pools can maintain iron balance, while in pathological cases, Fe^2+^ accumulates inside the cells and produces Fenton reactions, generating large amounts of ROS. In the presence of intracellular ROS, polyunsaturated fatty acids (PUFAs) undergo oxidation to produce lipid peroxides, which eventually cause cell ferroptosis.^17^ Since ferroptosis is mediated by intracellular iron overload, either increased iron absorption or decreased iron storage can affect the sensitivity of cells to ferroptosis. Therefore, understanding its underlying molecular and cellular mechanisms may open new avenues for the regulation of ferroptosis.

### 2.2 Amino acid metabolism

Ferroptosis depends heavily on amino acids. Directly inhibiting GPX4 and inhibiting glutathione (GSH), a cofactor for GPX4 function, can trigger ferroptosis. As the substrate of GPX4, GSH participates in the antioxidant system formed by GPX4 and the membrane protein system X_c_^−^ in the cell, which is a key factor affecting the occurrence of ferroptosis, and the synthesis of GSH is inseparable from the metabolism of amino acids. As an electron donor, the synthesized GSH can reduce toxic phospholipid peroxides to the corresponding alcohols under the action of GPX4, and simultaneously generate oxidized glutathione (GSSG). Indirect failure of GPX4 to function by depleting GSH can also lead to ferroptosis. As a result, GSH affects GPX4 catalytic efficiency and ferroptosis sensitivity in cancer cells.

The cystine transporter protein system X_c_^−^ on the cell membrane surface mediates the 1 : 1 exchange of intracellular glutamate and extracellular cystine, transporting cystine into the cell and generating cysteine through reducing reactions for GSH synthesis.^18^ Inhibiting the imbalance of amino acid metabolism caused by system X_c_^−^ can lead to ferroptosis, and glutamate itself can also affect the function of system X_c_^−^. A high extracellular glutamate concentration inhibits system X_c_^−^ and induces ferroptosis. Decreased cystine levels ultimately inactivate GPX4 by depleting GSH, and trigger ferroptosis.^19^ So, it is of great significance to search for the key molecules in ferroptosis (drugs in the process of GSH metabolism) for the study and utilization of ferroptosis.

### 2.3 Lipid metabolism

Phospholipids containing PUFAs are major substrates for ferroptosis lipid peroxidation.^20^ During this process, free PUFAs are esterified to become membrane phospholipids, and further oxidation induces ferroptosis. The increase in intracellular PUFAs enhances their susceptibility to ferroptosis. Studies have shown that phosphatidylethanolamine, including arachidonic acid and its derivative adrenal acid, is the key phospholipid in this process. Acyl-CoA synthase long-chain family member 4 (ACSL4) catalyzes the combination of free arachidonoyl (AA) or adenoyl (AdA) with coenzyme A (CoA) to form derivatives AA-CoA or AdA-CoA, which are then esterified by lysophosphatidylcholine acyltransferase 3 (LPCAT3) to phosphatidylethanolamine (PE). Therefore, ferroptosis susceptibility can be increased not only by supplementation with AA or other PUFAs, but also by inhibiting the activities of ACSL4 and LPCAT3.^21^ Upregulation of ACSL4 is considered a biomarker and contributor to ferroptosis.^22^ The generation of ferroptosis signals requires the formation of CoA derivatives of PUFAs and their combination with phospholipids, serving as potential targets for the treatment of ferroptosis-related diseases.

## 3. Small molecule inducers of ferroptosis

Ferroptosis is originally defined by a group of small molecules that induce selective death of tumour cells harboring RAS mutations.^11^ Stockwell and colleagues took advantage of the hypermutability of the RAS family of small GTPases to discover two novel RSL small molecules, RSL3 and erastin.^5^ Erastin is a prototypical ferroptosis inducer that reduces GSH levels by directly inhibiting system X_c_^−^. The activation of the Raf/MEK/ERK signaling pathway plays an important role in the process of erastin-triggered ferroptosis of tumour cells carrying RAS. The mechanism of action of RSL3-induced ferroptosis is to directly target GPX4. RSL3 target enzymes with nucleophilic sites (*e.g.* cysteine, serine, selenocysteine, *etc.*) and directly inactivate GPX4 through alkylation of selenocysteine.

Ferroptosis can be induced by both inhibitors without significant changes in cell size and nuclear morphology or apoptosis-like biochemical processes.^7^ Other studies have found that sulfasalazine and sorafenib trigger ferroptosis by inhibiting system X_c_^−^ in a similar manner to erastin. As a combination therapy, sulfasalazine enhances the effects of other chemotherapy drugs on gliomas. The results of the lethal activity analysis of various sorafenib analogs further suggest that there may be two potential mechanisms for the inhibition of system X_c_^−^ by sorafenib: (1) inactivation of kinases necessary for system X_c_^−^ activity; (2) interactions with non-kinase targets that have binding sites similar to sorafenib-sensitive kinases.

GSH controls the ferroptosis process by regulating the function of GPX4, which is essential for preventing harmful phospholipid oxidation. Insufficient supply of GSH will directly affect the function of GPX4, thus causing ferroptosis in cells. In this context, RSL3 (ref. 19) and FIN56 (ref. 23) are found to inactivate GPX4 and directly induce the accumulation of lipid peroxide, resulting in ferroptosis. FIN56 induces ferroptosis *via* two distinct pathways. First, FIN56 promotes the consumption and degradation of GPX4 under the catalysis of acetyl CoA carboxylase (ACC). Second, the endogenous antioxidant coenzyme Q10 (CoQ10) is depleted by the binding and activation of FIN56 to squalene synthase (SQS). This process enhances the sensitivity of cells to FIN56-induced ferroptosis. A number of other inducers of ferroptosis, such as DP12 (ref. 19), butylimine^24^ and cisplatin,^25^ may also cause synthetic lethality similar to GSH depletion (Table 2). Ferroptosis is closely related to cellular metabolism, as seen from the perspective of cellular metabolism.^26^ Given that ferroptosis is an iron-dependent process,^6^ modulation of iron metabolism and/or lipid metabolism can affect cell susceptibility to ferroptosis. Therefore, the targets of ferroptosis are rich and varied. It is often difficult to achieve satisfactory therapeutic effects for patients treated with small molecule compounds, which is also the root cause of the failure of most drug clinical trials so far.

**Table 2:** Small molecule inducers of ferroptosis

Ferroptosis inducers	Functional mechanism	Model systems
Erastin	Inhibit system X_c_^−^	HCC,^27^ GC,^28^AML,^29^ Calu-1,^4^ and HT-1080 (ref. 10)
RSL3	Inhibit GPX4 directly	HT-1080,^10^ 143B,^4^ and B16 (ref. 30)
Glutamate	Inhibit cystine uptake	HT-1080^4^ and HT-22 (ref. 31)
Sulfasalazine	Inhibit system X_c_^−^	HT-1080^4^ and B16 (ref. 32)
Sorafenib	Inhibit system X_c_^−^	HepG2^33^ and HT-29 (ref. 34)
FINO2	Direct oxidation of labile iron and inactivation of GPX4	HT-1080 (ref. 35)
FIN56	Degrade GPX4	BC^36^ and HT-1080 (ref. 37)
DPI2	GSH depletion	HT-1080 and BJ cell lines^19^
Butylimine	GSH depletion	NA
Cisplatin	GSH depletion	A549^38^ and A2780 (ref. 25)
Buthionine sulfoximine	GSH depletion	A549,^24^ 4T1,^39^ and HT-1080 (ref. 10)

## 4. Regulation of ferroptosis by natural compounds

For years, natural products, promising agents for drug development, have been extensively studied. A large number of substances separated from abundant natural resources is widely used in the prevention and treatment of various clinical diseases.^40^ Studies have discovered that many regulatory effects of natural compounds on iron metabolism and homeostasis are bound up with ferroptosis-related diseases. This section mainly summarizes various natural compounds that can induce ferroptosis, including saponins, terpenes, alkaloids, isothiocyanates and dibenzoyls (Table 3).

**Table 3:** Natural compounds regulating the ferroptosis pathway

Natural compounds	Source	Chemical structure	The way to regulate ferroptosis
Ruscogenin	*Ophiopogon japonicas*	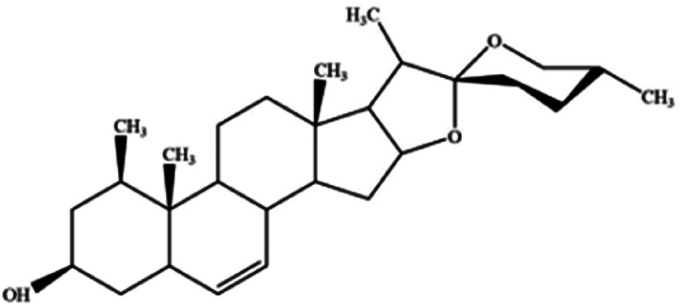	Increases intracellular ferrous iron and promotes ROS production
Albiziabioside A	*Albizia inundata*	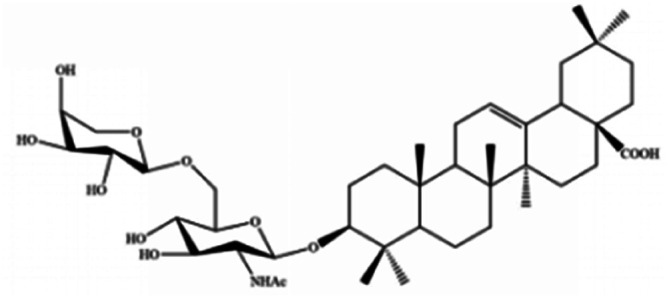	Activates p53 to induce ferroptosis
Artemisinin	*Artemisia annua* L.	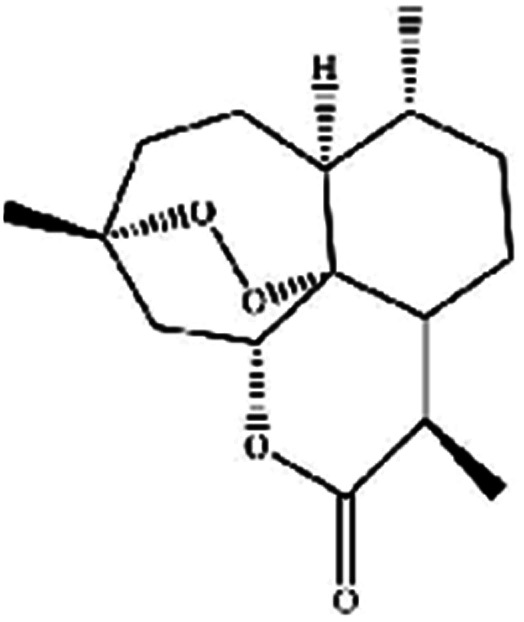	Trigges ROS overproduction and regulates the system X_c_^−^/GPX4 axis to induce ferroptosis
Artesunate		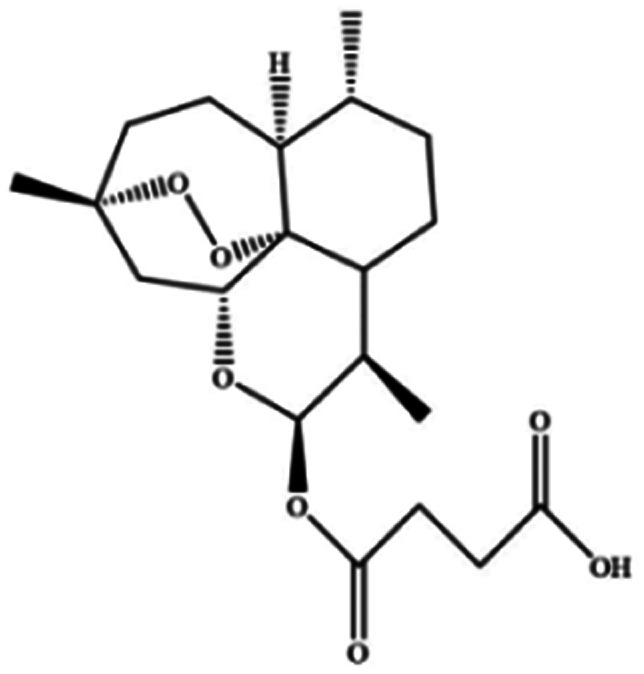	
Dihydroartemisinin		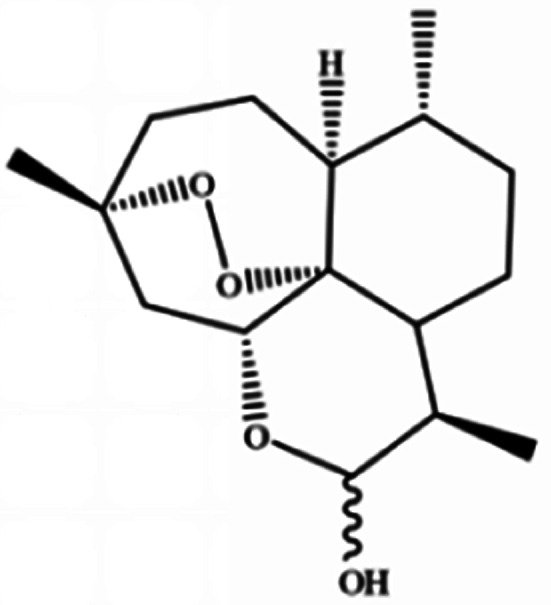	
β-elemene	*Curcuma wenyujin*	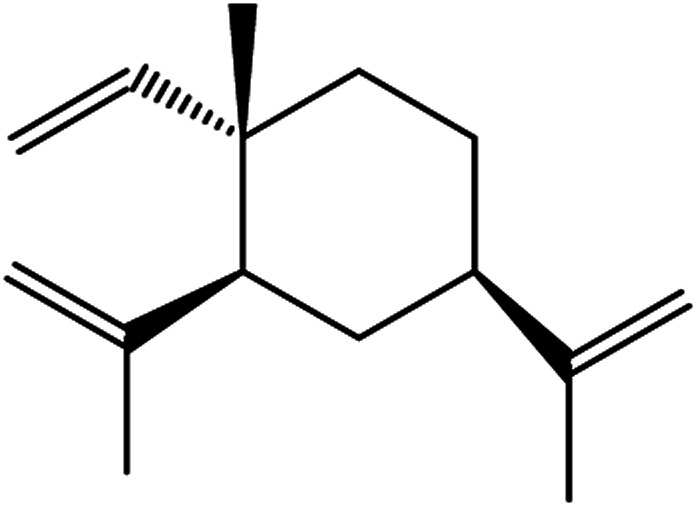	Combines with cetuximab treatment, up-regulation of transferrin, and down-regulation of GPX4, SLC7A11 and FTH1
Tanshinone II A	*Salvia miltiorrhiza* Bge	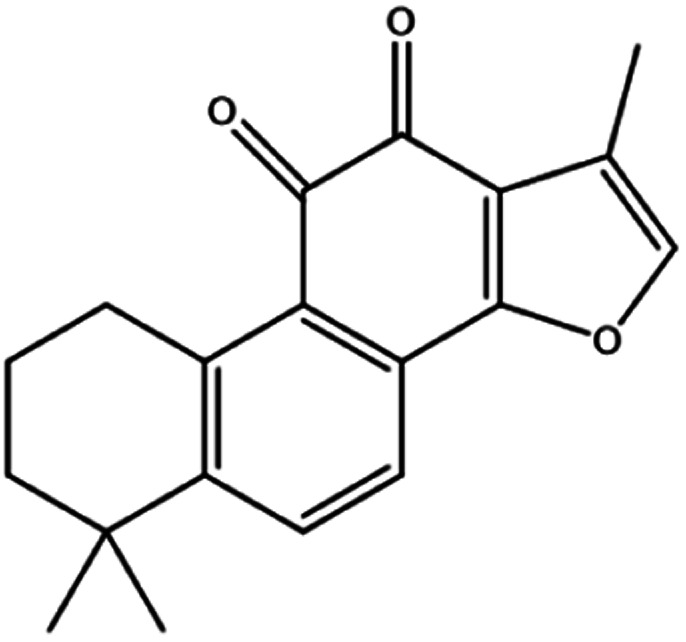	Induces ferroptosis *via* p53-mediated down-regulation of SLC7A11
Cryptotanshinone		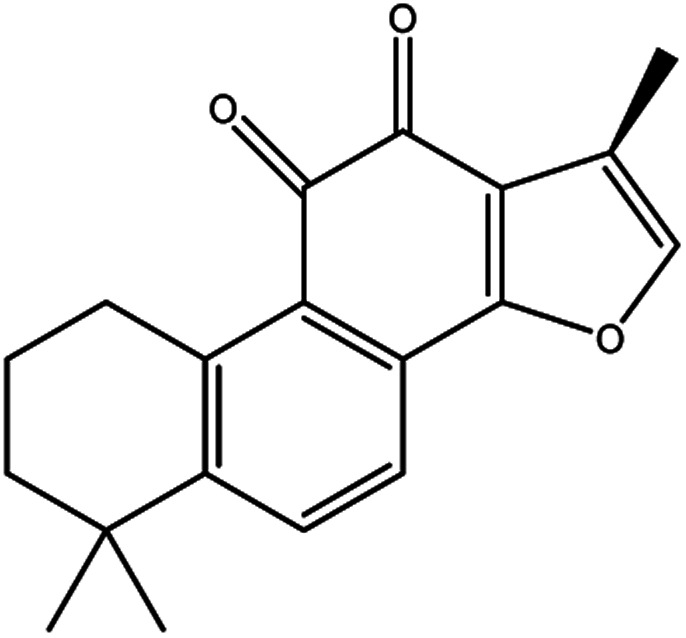	Up-regulates HSPB1 and GPX4 genes, and down-regulates IREB2 and VDAC2/3 genes
Piperlongumine	*Piper longum*	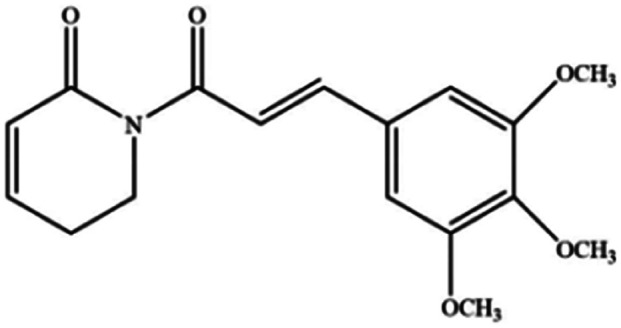	Increases ROS levels in tumour cells and decreases GSH levels which trigger ferroptosis
Ungeremine	Fruits and insects	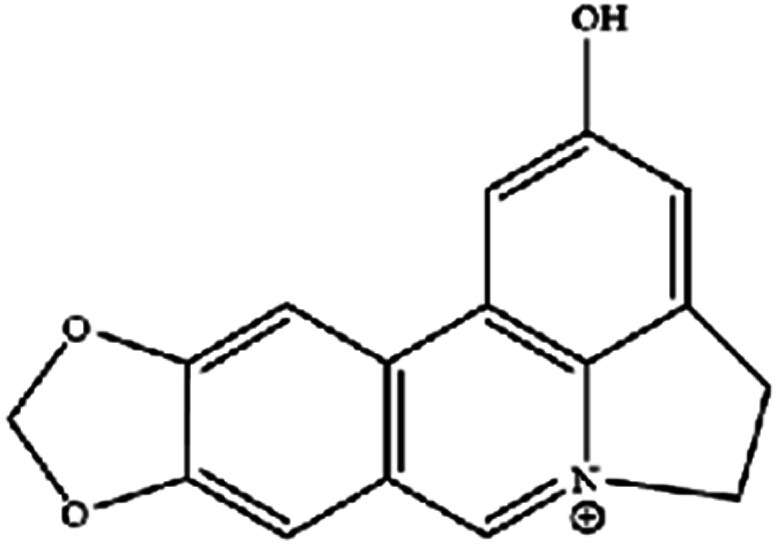	Unknown mechanism
Phenylethyl β-isothiocyanate	Green cruciferous vegetables	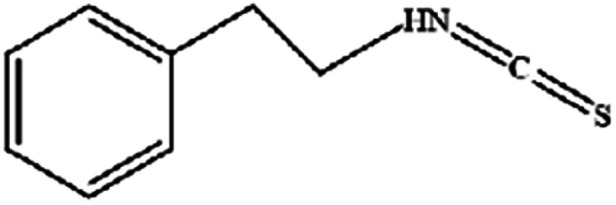	Regulates iron metabolism, inhibits GPX4 expression, activates ROS-MAPK signaling, and induces ferroptosis
Erianin	*Dendrobium chrysotoxum Lindl*	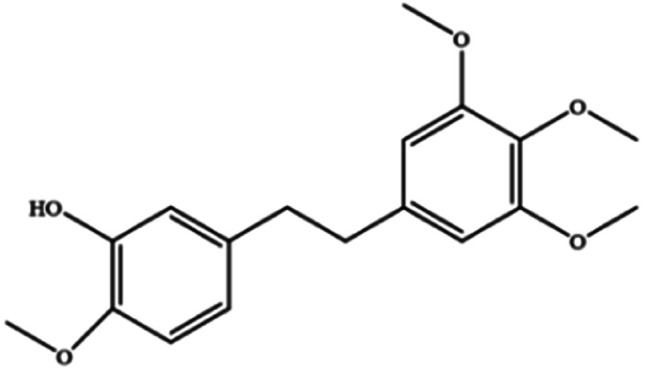	Elevates ROS and Fe^2+^ levels through the Ca^2+^/CaM pathway, ultimately leading to ferroptosis

The role of saponins in cancer therapy, prevention, and chemosensitization has been extensively studied. Saponins can trigger ferroptosis in tumour therapy, thereby exerting their therapeutic roles. For example, ruscogenin, a saponin found in *Ophiopogon japonicus*, induces ferroptosis, thereby inhibiting pancreatic cancer *in vivo* and *in vitro*.^41^ Ruscogenin activates ferroptosis by increasing intracellular ferrous content, promoting the production of ROS, and regulating the expression levels of transferrin and ferroportin. In colon cancer cells, albiziabioside A derivative compound D13 induces ferroptosis and cell death by activating p53.^42^ Also, albinoside A, conjugated to pyruvate dehydrogenase kinase inhibitors, inhibits the GPX4 pathway and induces ferroptosis through lipid peroxidation.^43^

Artemisinin and its derivatives have a long history in the application of traditional Chinese medicine. In Asia and Africa, they now top the list of treatments for malaria. In addition to antimalarial activity, an increasing number of studies have shown that artemisinin derivatives have various protective biological activities, and are widely used in the treatment of cancer, autoimmune diseases, inflammatory and pathogenic microorganism infectious diseases, and neurodegenerative diseases. For cancer treatment, there is evidence that artemisinin and its derivatives (such as artesunate, artemisinin, and dihydroartemisinin (DHA)) induce antioxidant stress in cancer cells by accumulating ROS, overexerting lipid peroxides and iron, and causing ferroptosis.^44^ DHA, a semisynthetic derivative of artemisinin, induces ferroptosis in leukemia cells and head and neck cancer cells.^45^ Recently, β-elemene was shown to be a ferroptosis inducer that sensitized KRAS-mutated colorectal cancer cells to the chemotherapeutic agent cetuximab.^46^

The tanshinone extract of *Salvia miltiorrhiza* Bge contains more than 10 monomers, of which tanshinone IIA (tanshinone IIA, Tan IIA) and cryptotanshinone (cryptanshinone, CTS) have active and high content. Guan *et al.*^47^ reported that Tan IIA could induce the up-regulation of p53 protein expression in BGC-823 and NCI-H87 gastric cancer cells, while the p53 protein could bind to the promoter region of SLC7A11 and inhibit its transcription, thereby inducing ferroptosis and inhibiting the proliferation of gastric cancer cells. CTS, an active ingredient of *Salvia miltiorrhiza* Bge, could inhibit the proliferation of A549 cells and cisplatin-resistant cells A549/DDP, and make A549/DDP more sensitive. CTS caused changes in ferroptosis-related genes, such as the up-regulation of HSPB1 and GPX4 mRNA expression of A549, and the down-regulation mRNA expression of IREB2 and mitochondrial voltage-dependent anion channel 2/3 (VDAC2/3), indicating that CTS anticancer effects were associated with ferroptosis.^48^

Piperlongumine, also known as piplartine, is an amide alkaloid derived from *Piper longum* that has been extensively studied for its excellent anticancer properties. In addition to inducing ROS-dependent ferroptosis in human pancreatic cancer cells, piperlongumine also enhances chemotherapeutic sensitivity.^49^ It is worth noting that a recent study clearly showed that ungeremine can induce ferroptosis and cell death in drug-resistant cancer cells through an unknown mechanism.^50^

The anticancer effects of phenylethyl β-isothiocyanate (PEITC) have been widely documented, and ferroptosis is another mechanism leading to cell death.^51^ Studies have found that PEITC alters iron metabolism, disturbs redox balance, activates ROS-MAPK signaling to induce ferroptosis and leads to human osteosarcoma cell death. Recent studies have demonstrated that Ca^2+^/CaM signaling was a key mediator of erianin-induced ferroptosis. Erianin exerted its anticancer effect by inducing Ca^2+^/CaM-dependent ferroptosis and inhibiting the proliferation and migration of lung cancer cells.^52^ It is speculated that erianin can target calmodulin to activate Ca^2+^ signal transduction and eventually lead to ferroptosis by increasing Ca^2+^ and Fe^2+^ levels.

It has been widely reported that natural bioactive components have a modulating effect on ferroptosis. Therefore, it is of great significance to accurately understand the potential relationship between their chemical structure and the corresponding ferroptosis regulation function. These phytochemicals should, however, be treated with caution when defining them as novel ferroptosis regulators. There should be clarification regarding whether these mechanisms directly affect core regulators such as ACSL4, SLC7A11, and GPX4, or whether they merely indirectly regulate ferroptosis-related metabolic products such as cysteine and glutamate. The exact regulatory targets of natural compounds, the molecular mechanism of regulation of ferroptosis and the structure function relationship are still unclear, and need to be further discussed.

## 5. Progress of nanomaterials in targeting ferroptosis

With the deepening of research on the mechanism of ferroptosis, the anti-cancer treatments of nanomaterials targeting ferroptosis have made good progress. Although the research of anti-cancer methods based on nanotechnology has exploded, the targeting efficiency, therapeutic results and clinical transformation of these nanoplatforms are far from satisfactory.^53,54^ At present, most of the other nanomaterials are mainly used for preclinical research, except for the intravenous iron oxide preparations of Ferumoxytol and Cornell dots, which are used for clinical settings. Therefore, it is necessary to have a deeper understanding of complex biosystem and nano-biological interactions. Only in this way can we discover if nanomedicine has become a new application direction of ferroptosis. Iron-based and non-iron-based nanomaterials are the two general categories for nanomaterials that target ferroptosis. In this section, we will focus on the current situation and application of the above two nanomaterials targeting ferroptosis.

### 5.1 Iron-based nanomaterials

**Fig. 2:**
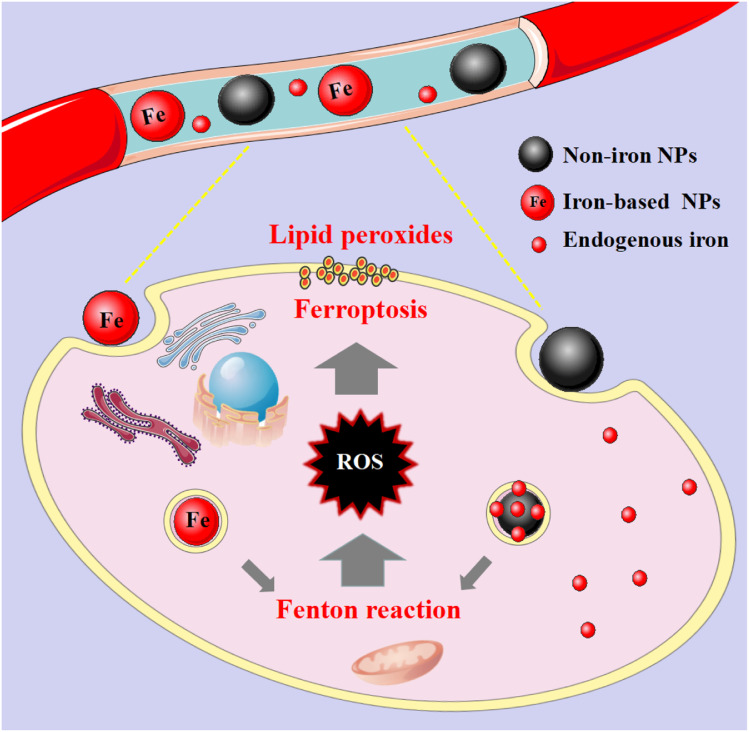
Mechanistic illustration of iron- and non-iron-based nanomaterials for ferroptosis-based cancer therapy. After these nanomaterials are endocytosed into cells, they release their own iron or endogenous iron under the acidic conditions of lysosomes, and participate in the Fenton reaction to generate excess ROS, which lead to lipid peroxidation.

These nanomaterials have the superiority of directly triggering the Fenton reaction due to the presence of iron. As the name implies, such nanomaterials contain iron, and their general consequence is to increase the availability of iron in cells. Although the exact and complete role of iron in ferroptosis is unknown, iron-catalyzed ROS production (Fenton reaction) is currently believed to be an important pathway for ferroptosis. Since tumour cells are prone to ferroptosis, iron-based nanomaterials can be used as potential drugs for the treatment of tumours. They possess the characteristics of nanomaterials themselves, which can specifically aggregate at tumour sites through passive and active targeting, and iron can be released into acidic lysosomes as Fe^2+^ or Fe^3+^ ions, participate in the Fenton reaction to generate ROS to induce ferroptosis and kill tumour cells (Fig. 2). And it can be combined with other anti-tumour properties or diagnostic properties of the nanoparticles themselves for tumour treatment or integration of diagnosis and treatment. Common iron-based nanomaterials mainly include the following categories:

#### 5.1.1 Iron oxide NPs (IO NPs)

IO NPs are representative iron-based nanomaterials, which have been approved by the US FDA for intravenous treatment of clinical iron deficiency. IO NPs mainly kill cells by releasing free iron to generate a large amount of ROS. The following will be elaborated in two parts: clinically approved products and preclinical research.

##### 5.1.1.1 Clinically approved products

Recently, IO NPs have been shown to induce ferroptosis in tumour cells. Zanganeh *et al.*^55^ found that iron oxide nanoparticles (ferumoxytol) can not only regulate intracellular iron content, but also make macrophages differentiate into the M1 type. Differentiated M1 macrophages can produce a large amount of H_2_O_2_, which can react with iron ions in the body to generate ROS through the Fenton reaction, and promote the occurrence of ferroptosis. *In vivo*, the nanoparticles significantly inhibited the growth of subcutaneous adenocarcinoma in mice and prevented the progression of liver metastasis. According to previous studies,^56^ proinflammatory M1 macrophages in wounds can release H_2_O_2_, and it can be speculated that H_2_O_2_ released by macrophages and Fe^3+^ or Fe^2+^ in ferumoxytol produce highly toxic ROS, which finally reaches the effect of tumour cell growth inhibition.

US FDA-approved Cornell dots (ultrasmall poly[ethylene glycol]-coated silica nanoparticles) induce ferroptosis and suppress tumour growth. Kim and colleagues were the first to discover that nanoparticles can cause ferroptosis. By structuring αMSH-PEG-C′ dots with near-infrared (NIR) fluorescent ultra-small silica nanoparticles (C′ dots), polyethylene glycol (pegylated C′ dots), and alpha-melanocyte-stimulating hormone (α-MSH), they stimulated ferroptosis in tumour cells and observed an increase in the Fe amount, an enrichment of ROS, and a reduction in the GSH content.^57^*In vitro* and *in vivo* experiments demonstrated the effectiveness of α-MSH-PEG-C′ dot-induced ferroptosis in various cancer models.

##### 5.1.1.2 Preclinical research

Furthermore, this nanomaterial can be further modified and engineered for better ferroptosis induction or tumour targeting. Generally speaking, the intention of this expansion is to endow the system with imaging diagnostic functions, or to achieve better therapeutic effects by promoting the Fenton reaction to generate more ROS. For example, Zhou *et al.* developed an activatable system enabling tumour-specific singlet oxygen (^1^O_2_) generation for cancer therapy, based on the Fenton-like reaction between linoleic acid hydroperoxide (LAHP) tethered on iron oxide nanoparticles (IO NPs) and the released iron(ii) ions from IO NPs under acidic-pH conditions. The results demonstrated that engineered IO-LAPHNPs induced tumour cell death *in vitro* and *in vivo via* singlet oxygen generation and ROS-mediated ferroptosis, ultimately inhibiting tumour growth in tumour-bearing mice after intravenous IO-LAHP NPs.^58^

In addition, Li *et al.* successfully prepared a H_2_O_2_-filled polymersome. Liquid H_2_O_2_ was encapsulated in the hydrophilic core in the polymer, while Fe_3_O_4_ nanoparticles were filled in the shell. The polymersome could be used in ultrasound imaging diagnostic systems and ROS-mediated cancer therapy.^59^ When exposed to ultrasound, the polymeric vesicles were easily destroyed, generating ·OH through the Fenton reaction of H_2_O_2_ and Fe_3_O_4_. The results demonstrated that malignant tumours could be completely removed in a non-thermal process. Another study integrated native glucose oxidase (GOD) and ultrasmall Fe_3_O_4_ nanoparticles into dendritic mesoporous silica nanoparticles (DMSNs) to prepare biodegradable and sequentially functional GOD-Fe_3_O_4_@DMSN (GFD) nanocatalysts with high tumour specificity and therapeutic efficacy. In tumour tissue, GOD could catalyze glucose to generate H_2_O_2_, and then interact with Fe_3_O_4_ nanoparticles through the Fenton reaction to produce highly toxic ·OH. The accumulation of highly toxic ·OH would eventually lead to the occurrence of ferroptosis, and intravenous injection into mice could effectively inhibit tumour growth.^60^

**Fig. 3:**
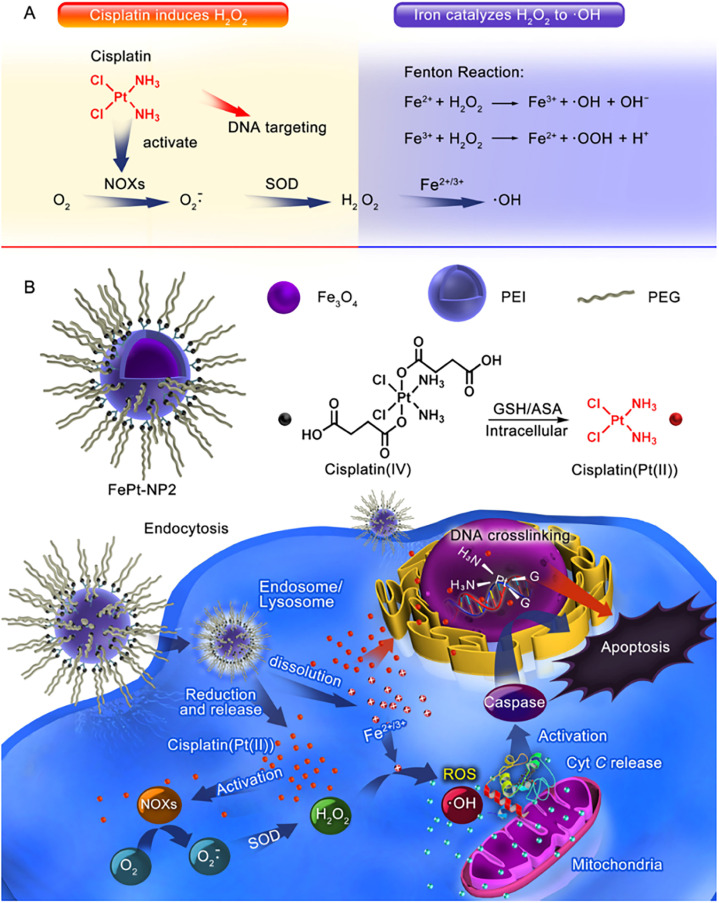
(A) Cisplatin could activate NOX and catalyze O_2_ to form superoxide and H_2_O_2_; iron ions converted H_2_O_2_ to ·OH through the Fenton reaction. (B) Iron oxide nanoparticles containing cisplatin(iv) prodrug (FePt-NP2) could activate NOXs and catalyse O_2_ to form H_2_O_2_ by releasing cisplatin. H_2_O_2_ and iron ions generated excess ROS through the Fenton reaction, resulting from rapid lipid and protein oxidation and DNA damage, and triggering the ROS/CytC/caspase-3 pathway leading to apoptosis. Reproduced with permission.^61^ Copyright 2017, American Chemical Society.

Furthermore, Ma *et al.*^61^ successfully constructed an IO NP platform loaded with cisplatin(iv) prodrug (FePt-NP2) (Fig. 3). The sequential drug delivery system had the advantages of high drug bioavailability, MIR-guided tumour targeting, and enhanced therapeutic effect of cisplatin. The nanocarrier promoted the internalization of cisplatin and iron by tumour cells, and the cisplatin(iv) prodrug could be rapidly reduced to toxic cisplatin followed by the formation of Pt–DNA complexes, which activated NOXs and formed H_2_O_2_. Iron nanoparticles were degraded and metabolized in cancer cells, releasing unstable iron ions, and the Fenton reaction catalyzes the formation of ROS from H_2_O_2_, leading to the oxidation of cell membranes. The abundant intracellular ROS worked synergistically with cisplatin to exert an enhanced anti-tumour effect through three major pathways including ROS/CytC/caspase-3.

#### 5.1.2 Iron-based upconversion NPs

**Fig. 4:**
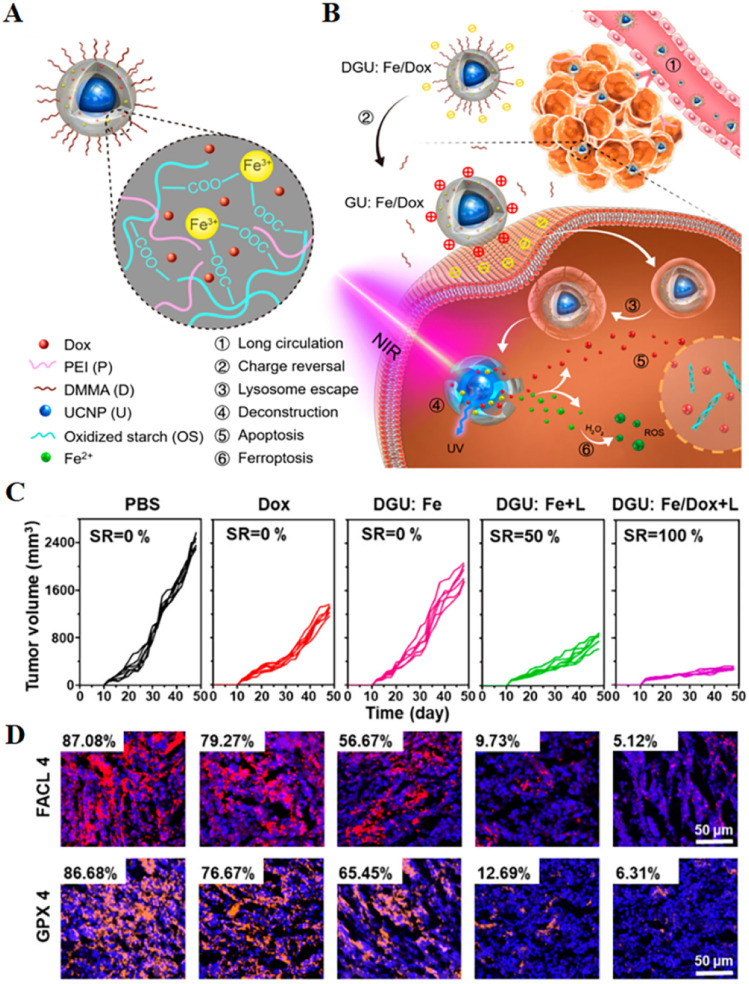
(A and B) Schematic diagram of the structure of a nanolongan and its anticancer mechanism. Nanolongan included inner UCNPs and an outer gel shell. The shell structure was formed by the coordination of Fe^3+^ with the carboxyl groups of the oxidized starch polymer and wrapped with PEI and DMMA. The chemotherapeutic drug Dox was successfully loaded within the shell. The specific uptake pathway of DGU:Fe/Dox nanolongan: DGU:Fe/Dox nanolongan nanoparticles aggregated at the tumour site by passive targeting (EPR effect). Due to the acidic conditions of the tumour micro-environment, the DMMA molecules on the surface of the nanolongan fell off, thereby exposing the PEI molecules, and the charge changed from negative to positive. The proton sponge effect enabled the nanolongan to successfully escape from the lysosome, and then under NIR irradiation, the energy transfer within the UCNP resulted in the deconstruction of the outer shell. Thereby, the anticancer drug Dox and the ROS generated by the Fe^2+^-triggered Fenton reaction were released to synergistically induce apoptosis and ferroptosis. (C) Growth inhibition of 4T1 tumours treated with different formulations after 48 days. (D) Immunohistochemical images of FACL4 and GPX4 in tumour tissues. Reproduced with permission.^64^ Copyright 2019, American Chemical Society.

The efficiency of ROS generation from the Fenton reaction involving iron ions can be accelerated by UV/visible light irradiation; however, UV light has low penetration and rapid decay in tissues, which limits its application.^62^ Long wavelength near-infrared light, which has the advantages of less biological tissue damage and significant tissue penetration depth, can be converted into short wavelength ultraviolet/visible light *via* upconversion nanoparticles (UCNPs). Based on the above theory, iron-based UCNPs can enhance the efficacy of ferroptosis by enhancing the Fenton reaction system. Therefore, Hu *et al.*^63^ synthesized iron-based UCNPs, a phototherapy strategy for inducing the generation of ROS by triggering the Fenton reaction in mitochondria through long-wave near-infrared light irradiation. In this study, NIR light converted UV/vis light *via* UCNPs was used to catalyze the intramitochondrial Fenton reaction between the delivered Fe^2+^ and H_2_O_2_ species over-expressed in the mitochondria of cancer cells to kill cancer cells *in situ*. Another study designed a nanolongan delivery system with the typical structure of one core (UCNPs) in one gel particle (Fe^3+^ cross-linked oxidized starch) with multiple on-demand conversions (Fig. 4).^64^ In this system, with further near infrared light irradiation, the UCNPs featured with NIR to ultraviolet light (UV) conversion enabled the reduction of Fe^3+^ to Fe^2+^. This valence switch deconstructed the gel network, enabling rapid release of the drug pair. As a result, Fe^2+^ reacted with H_2_O_2_ in the cytoplasm to generate ROS leading to ferroptosis, while Dox diffused into the nucleus to induce apoptosis. The results of *in vivo* experiments found that DGU:Fe/Dox + L could significantly reduce the expression levels of GPX4 and FACL4, thereby achieving superior anticancer effects. These remarkable features clearly revealed that this nanolongan with multiple on-demand transformations could be used for effective ferroptosis-apoptosis combined anticancer therapy.^64^

#### 5.1.3 Amorphous iron nanoparticles (AFe NPs)

These nanomedicines are often made of amorphous iron and nanoparticles. They can usually rapidly ionize and release mixed iron in the acidic tumour micro-environment to increase the sensitivity of cells to ferroptosis, so as to kill cancer cells more effectively. In one study, Zhang *et al.*^65^ presented that AFe NPs reacted with H_2_O_2_ in tumour cells to produce ROS for cancer specific treatment without an external energy input. *In vitro* studies showed that cell viability was significantly reduced in the presence of AFe NPs and H_2_O_2_ at pH = 6.5 compared to controls at pH = 7.4. With the increase of the concentration of AFe NPs, the inhibition of cell viability became more obvious.

AFe NPs are found to rapidly ionize in acidic tumours to release Fe^II^ on demand and enable subsequent localized Fenton responses for specific cancer treatments. Owing to the stimulus-responsive nature of this process, the therapy is highly specific. In another important study, Liu *et al.* developed a unique RNAi platform of amorphous iron oxide (AIO) nanoparticles for combined cancer treatment by simultaneously silencing MCT4 and enhancing ROS. On the one hand, NP-mediated silencing of MCT4 could block intracellular lactate/H^+^ efflux, leading to acidosis induced tumour cell death. On the other hand, AIO NPs responded to the acidic pH after cellular uptake, and the released iron ions reacted with H_2_O_2_ to generate highly reactive and toxic ·OH *via* a Fenton-like reaction. More importantly, MCT4 silencing could inhibit the lactic acid efflux in cells, and further promote the production of H_2_O_2_, thereby enhancing ferroptosis, which had an effective combination therapy.^66^

#### 5.1.4 FePt nanoparticles

In recent years, chemically disordered face centered cubic (fcc) iron platinum nanoparticles (FePt NPs), as particular nanomaterials with intrinsic magnetism, have attracted more and more attention and research in the fields of information storage, biosensors, biological separation and biological imaging.^67^ In addition, Pt/Fe elements are two excellent contrast agents for CT and T2-weight MR imaging, giving FePt NPs the ability to simultaneously achieve imaging diagnosis. It has broad development prospects in the field of biological applications. Yue *et al.*^68^ examined a pH-responsive multifunctional therapy method, which could successfully achieve dual-modality MRI/CT imaging and suppression of carcinoma *in situ*. Due to the low pH value of tumour cells, FePt NPs would release highly active Fe ions, which could catalyze the decomposition of H_2_O_2_ into ROS within the cells, further inducing cancer cell ferroptosis. Conjugated with folic acid (FA), FePt-dimercaptosuccinic acid/PEGylated graphene oxide-folate (FePt-DMSA/GO-PEG FA) composite nanoassemblies (FePt/GO CNs) could effectively target FA receptor-positive tumour cells showing significant toxicity, but no significant toxicity was shown to FA receptor-negative normal cells. The cytotoxicity of FePt-based nanocomposites, as determined by WST-1, showed significant toxicity against MCF-7, HeLa and HepG2 cells with corresponding half-maximal inhibitory concentrations (IC_50_) of approx. 40, 52, and 47 μg mL^−1^, respectively. At the same time, the decomposition of FePt could dramatically reduce the T2-weighted MRI signal and increase the ROS signal, so that it could monitor the release of Fe in tumour cells in real time and *in situ*. In another work, an acid-sensitive nanotherapeutic (FePt@MnO)@DSPE-PEG5000-FA (FMDF NPs) was successfully constructed for MR imaging-guided cancer ferroptosis chemodynamic therapy (FCDT). FMDF NPs could specifically target FA receptor-positive tumour cells (HeLa, *etc.*), and the active Fe^2+^ released from the system converted intracellular H_2_O_2_ into ROS through the Fenton reaction, thereby inducing ferroptosis. Furthermore, the high concentration of Mn^2+^ ions released from the MnO domains enhanced T1/T2-weighted MR imaging. FMDF NPs could significantly enhance the contrast distinction of dual-mode MR/CT imaging between tumours and surrounding tissues for accurate real-time monitoring of the tumour location. Furthermore, *in vivo* anticancer studies showed that the growth of solid tumour models was significantly inhibited after treatment with FMDF NPs, with no obvious damage to other major organs.^69^

#### 5.1.5 Iron–organic frameworks

**Fig. 5:**
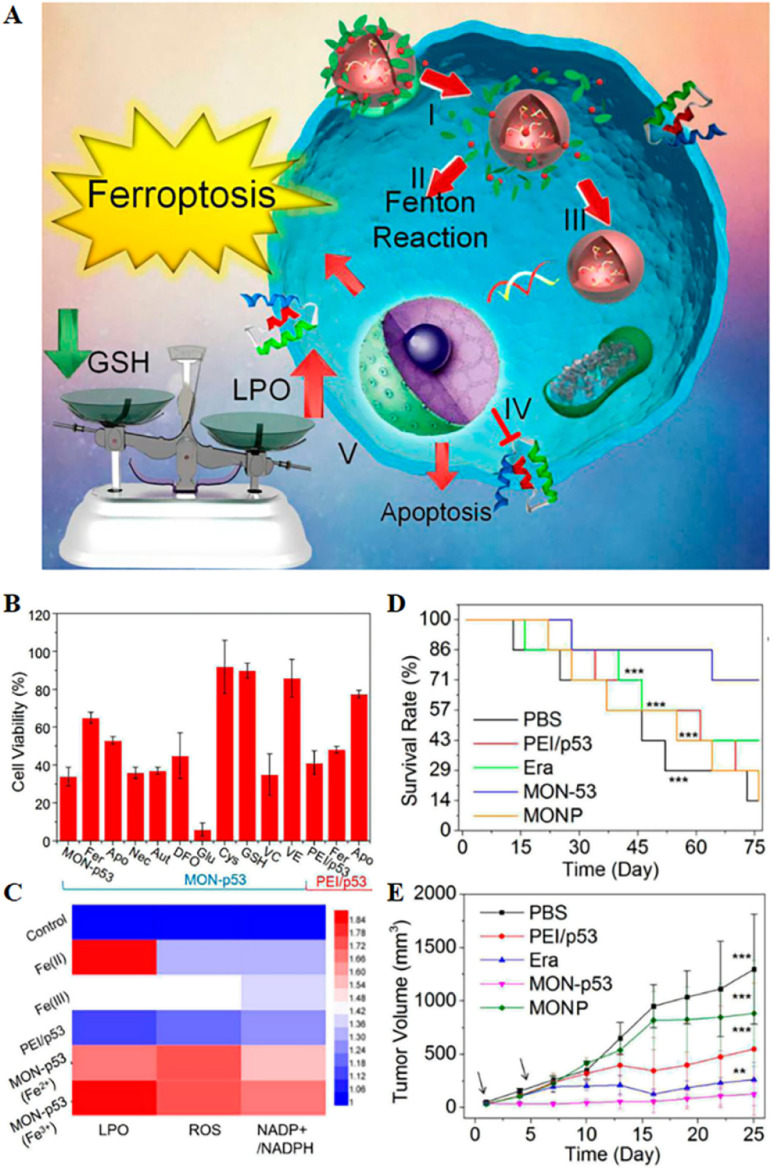
(A) Schematic representation of MON-p53 anticancer therapy. (I) Endocytosis of MON-p53. (II) MON-triggered Fenton reaction. (III) Transfection and expression of the p53 protein. (IV) Inhibitory effect of the p53 protein on transmembrane protein SLC7A11. (V) The Fenton reaction led to LPO accumulation, and suppression of SLC7A11 expression resulting in GSH depletion. The p53 protein was involved in the apoptotic pathway and triggered apoptosis. (B) Viability of HT1080 cells after treatment with different preparations. (C) LPO, ROS and NADP^+^/NADPH contents of HT1080 cells treated with different formulations. (D) Survival curves of HT1080 tumour-bearing mice in different treatment groups. (E) Tumour volume curve of HT1080 tumour-bearing mice for 25 consecutive days. Reproduced with permission.^70^ Copyright 2016, American Chemical Society.

These nanomedicines mainly include two categories: organic network nanoparticles and organic framework nanoparticles. Metal–organic frameworks have been widely used in various biomedical applications in recent years due to their facile synthesis, low cost, high biocompatibility, high versatility, ultra-high porosity (up to 90% free volume) and huge internal surface area. Metal polyphenol networks can be formed by coordinating transition metal ions with catechol structures in polyphenols. Inspired by this, Zheng *et al.*^70^ combined the FDA-approved food additive tannic acid extracted from green tea with iron ions to form MON on the surface of a polyethyleneimine/p53 plasmid complex (PEI/p53) to obtain MON-p53, and MON coating was used to enhance the ferroptosis-inducing ability of p53 (Fig. 5). The results showed that the obtained MON-p53 could clear tumour cells through a mixed ferroptosis/apoptosis pathway. A 75 day anticancer experiment showed that MON-p53 could not only inhibit tumour growth and lung metastasis due to its strong intracellular oxidative stress ability, but also prolong the lifespan of tumour-bearing mice. As MON-p53 became internalized, ferric ions could induce Fenton reactions, generating ROS in biofilms, causing severe lipid peroxidation. Additionally, the p53 protein suppressed lipid peroxide elimination. The above two mechanisms together increased the ferroptosis sensitivity induced by MON-p53, indicating the direction for the design of anti-cancer nanomaterials.

**Fig. 6:**
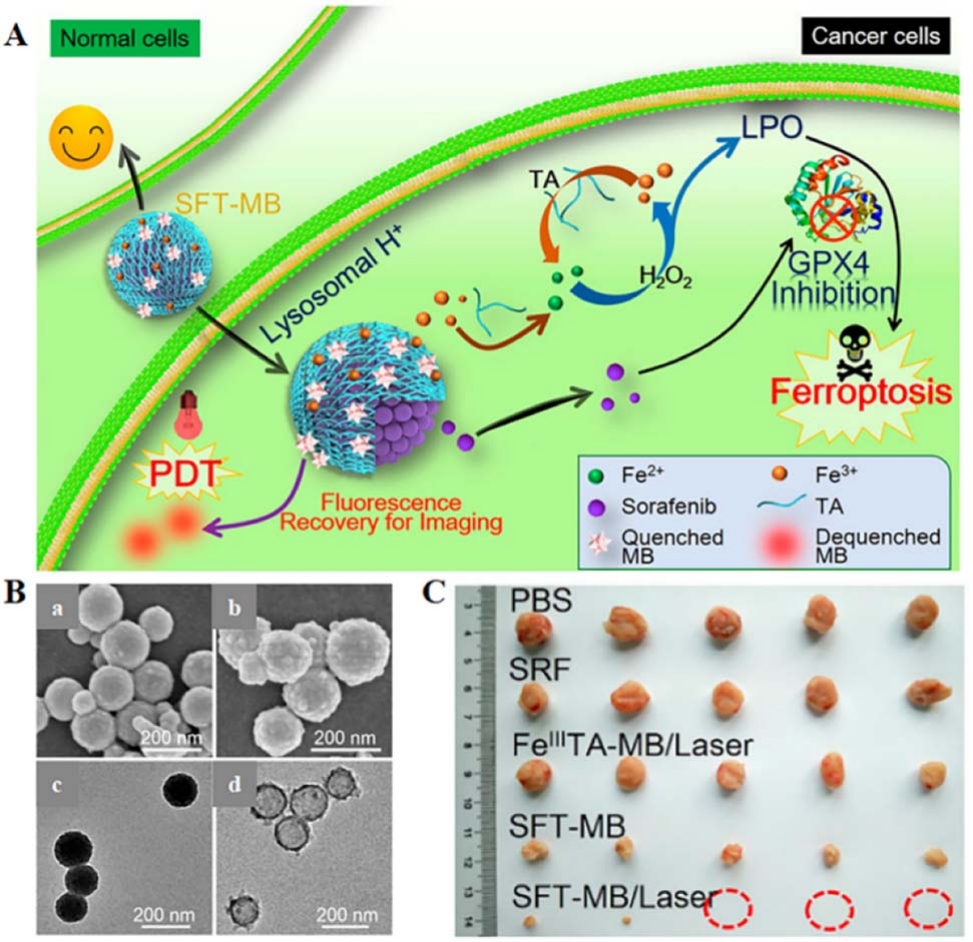
(A) Schematic diagram of the combination of SFT-mediated ferroptosis and image-guided PDT in the treatment of tumours. Due to the acidic environment within the lysosome, allowing the responsive release of SRF and TA, SRF could trigger ferroptosis by inhibiting the activity of the GPX4 enzyme. The role of TA was to participate in redox reactions, reducing Fe^3+^ to Fe^2+^ to produce lipid peroxides that participate in ferroptosis. Sustained Fe^2+^ supply produced long-term cytotoxicity, whereas SFT had negligible toxic effects on normal cells. (B) SEM images of SFT (a) and SFT-MB (b); TEM images of SFT (c) and DMF-etched (d). (C) Tumour photographs obtained after 14 days of treatment in different treatment groups. Reproduced with permission.^71^ Copyright 2018, American Chemical Society.

Inspired by the industrial electro-Fenton technology featuring electrochemical iron cycling, iron supply regeneration nanoengineering was constructed to intervene in tumour iron metabolism to enhance ferroptosis.^71^ Fe^3+^ ions and naturally derived tannins (TA, an acidity-activated reducing agent) selfdeposited on sorafenib (SRF, ferroptosis-inducing agent) nanocrystals to form networked corona, thereby generating core-corona SRF@Fe^III^ TA (SFT) nanoengineering. In the presence of lysosomal acid, SFT nanoparticles underwent corona dissociation, allowing controlled release of SRF to inhibit GPX4 enzyme for ferroptosis initiation. At the same time, TA provided an iron redox cycle for a continuous supply of Fe^2+^, which helped in H_2_O_2_ production of lipid peroxides in overloaded tumour cells (Fig. 6). SFT could effectively kill tumour cells through apoptosis and ferroptosis.

The photosensitizer-adsorbed SFT exhibited rapid tumour imaging and complete tumour elimination due to acid-responsive fluorescence recovery. All the results suggested a potential ferroptosis-inducing nanotherapeutic for tumour imaging, imaging-guided PDT, and ferroptosis therapy with multiple functions.

The discovery of the mechanism of ferroptosis enables iron-based metal–organic frameworks to be used for efficient cancer-specific therapy. For example, Wang *et al.*^72^ used a layer-by-layer approach to design core–shell Mn_3_[Co(CN)_6_]_2_@MIL-100(Fe) metal–organic framework (CS-MOFs) nanocubes and loaded them with artesunate (AS) for cancer treatment. In the tumour micro-environment, the degradation of CS-MOFs enabled Fe(iii) and AS to be released on demand. Intracellular ferrous ions catalyzed AS to generate carbon center free radicals and ROS, further leading to cell death. Animal experiments showed that compared with free AS alone, the anti-tumour effect of CS-MOFs@AS was enhanced by 5.79 times, indicating that it was a potential integrated probe for tumour diagnosis and treatment.

#### 5.1.6 Iron-containing nanocatalysts

**Fig. 7:**
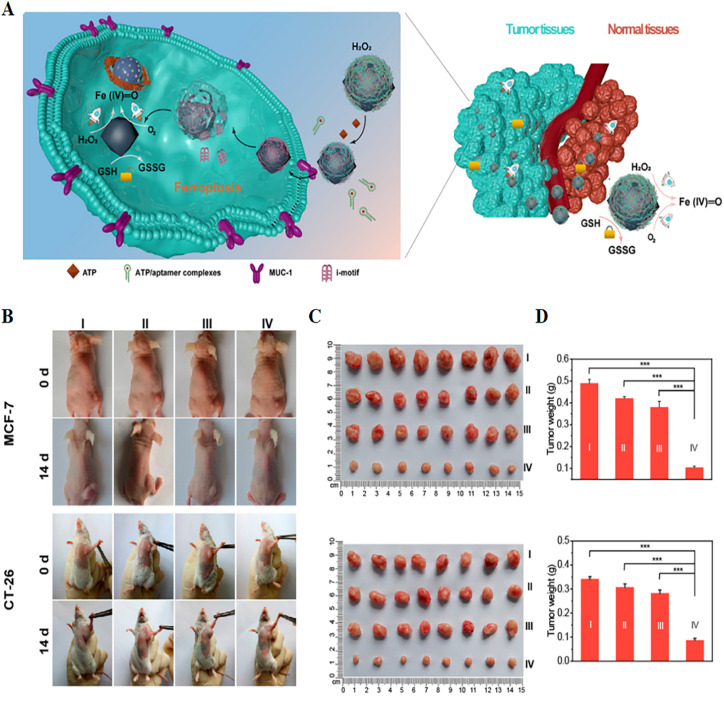
(A) Schematic illustration of a self-adaptive single-atom nanozyme and its specific killing effect on tumour tissue. (B) Photographs of tumour-bearing mice obtained before and after 14 days of treatment in different treatment groups. (C and D) Tumour photographs and weights obtained after 14 days of treatment in different treatment groups. I, control; II, Fe_3_O_4_; III, macDNA-Fe/PMCS + liproxstatin-1; IV, macDNA-Fe/PMCS. Reproduced with permission.^74^ Copyright 2022, American Chemical Society.

At present, researchers have developed various nanocatalysts to induce local Fenton reaction in tumours for anti-tumour therapy. Shi Jianlin's group^73^ developed a high-performance nanocatalytic system for inducing ferroptosis in tumour cells. The PEG-modified Fe-containing nanocatalysts (PSAF NCs) were constructed by dispersing elemental iron into nitrogen-doped carbon nanomaterials, followed by PEGylation of nanoparticles. According to the proton-mediated homolysis mechanism, H_2_O_2_ is easily absorbed and dissociated by amorphous atomic iron. In the acidic tumour micro-environment, PSAF NCs could effectively trigger the *in situ* Fenton reaction and generate toxic ·OH. The specific production of ·OH in tumour cells could lead to the rapid accumulation of lipid peroxides and induce ferroptosis and apoptosis. *In vivo* results showed that after 15 days of intravenous or intratumoural injection of PSAF NCs, the tumour inhibition rates of 4T1 tumour-bearing model animals were 40.01% and 63.49%, respectively. More importantly, the Fe-containing nanocatalysts exhibited good biodegradability and biocompatibility, and showed no obvious systemic toxicity. Cao *et al.*^74^ found a self-adaptive ferroptosis platform (macDNA-Fe/PMCS) by engineering single-atom nanozyme (SAzyme) surface-linked DNA modulators (Fig. 7). The modulator could not only specifically enhance the generation of ROS, but also enable SAzymes to have the ability to consume anti-tumour GSH on demand in tumour cells, thereby enhancing their affinity for cancer cells and improving their specificity and efficiency. The performance of this self-adaptive ferroptosis platform was validated in two models of colon cancer and breast cancer. *In vivo* results showed that the tumour growth of both tumour-bearing mice was significantly inhibited after 14 days of intraperitoneal injection of macDNA-Fe/PMCS. In addition, macDNAFe/PMCS could significantly reduce the high expression of GSH in tumours. This self-adaptive ferroptosis platform facilitated the development of selective cancer therapy.

### 5.2 Non-iron-based nanomaterials

These nanomaterials are iron-free and extremely diverse. Recent studies have reported a wide variety of non-iron nanomaterials for biomedical applications. For the first time, Kim *et al.* proved that non-iron based nanomaterials can also be developed for cancer treatment based on ferroptosis because they can load and transfer endogenous iron into cells.^57^ The research results have demonstrated that the deprotonated surface silanol groups and/or fractal internal structures of the natural silica particles can lead to the adsorption and/or incorporation of iron into their structures, thus inducing the occurrence of cell ferroptosis. Non-iron based nanomaterials can not only activate ferroptosis by promoting ROS production, but also achieve the goal of promoting ferroptosis by destroying the antioxidant defense system, accumulating lipid peroxides, and even participating in regulating cell metabolism. These nanomaterials improve their therapeutic efficacy by loading different ferroptosis inducers. Expanding PUFAs or using intracellular lipid proteins promotes the Fenton reaction. These nanomaterials also have relatively diverse triggering pathways. Besides, the disruption of the activity of iron receptors/channels in cell membranes may be the main reason for the promotion of ferroptosis by nanomaterials composed of other partially metal elements. Non-iron-based nanomaterials typically come in the following forms:

#### 5.2.1 Simple ferroptosis inducer (FIN) carrier system

The main ingredient of these nanomaterials is FIN, which triggers ferroptosis. Among them, the release of RSL3 from mPEG-PLys-AA/RSL3 triggers ferroptotic cancer cell death.^75^ RSL3 was encapsulated in micelles to target GPX4. In drug-resistant human ovarian adenocarcinoma cells, the cytotoxicity of RSL3 loaded micelles was 30 times higher than that of activatable control micelles due to the ferroptosis mechanism. Lipid peroxidation-induced reduction in intracellular GSH levels also contributed, which enhanced the potency of RSL3 to induce ferroptosis and rendered the drug-loaded micelles fully active. In addition, supramolecular interaction between erastin and Ce6 has been reported to construct a carrier-free nanodrug, namely, Ce6-erastin.^76^

Ferroptosis induced by erastin resulted in the accumulation of lipid-based ROS and demonstrated excellent cytotoxicity. Under light irradiation, Ce6-erastin showed unprecedented ability of oxygen self-sufficiency, ensuring effective oxygen dependent PDT. Sufficient O_2_ production could produce higher levels of ROS and cause oxidative damage to cellular components. The results showed that after laser irradiation, the excessive accumulation of ROS, the increase in O_2_ concentration and the inhibition of SLC7A11 expression in cells would increase the toxicity to CAL-27 cells. Furthermore, enhanced PDT combined with ferroptosis demonstrated good anti-tumour activity in xenograft tumour mice.

Recent studies have found that exosomes can be another important carrier for some ferroptosis inducers. An exosome-targeted delivery system loaded with erastin (erastin@FA-exo) was investigated by Yu *et al.*^77^ In comparison with free erastin, erastin@FA-exo could inhibit MDA-MB-231 cell proliferation and migration more effectively. Additionally, erastin@FA-exo promoted ferroptosis by consuming GSH and generating ROS. Meanwhile, another study found that a ferroptosis inducer (Erastin, Er) and photosensitizer (Rose Bengal, RB) could be effectively coated in exosomes to form drug-loaded exosomes (Er/RB@Exos^CD47^).^78^ Exos^CD47^ enabled the exosomes to effectively escape phagocytosis by a mononuclear phagocyte system, thereby increasing their distribution in tumour tissues. The results of *in vivo* and *in vitro* experiments showed that Er/RB@Exos^CD47^ strongly induced ferroptosis with minimized toxicity in the liver and kidney. Thus, the engineered exosomes provided a promising strategy for the treatment of malignant tumours.

#### 5.2.2 Photodynamic/sonodynamic nanomedicines

In addition to their active release mechanism, these nanomedicines can also be used as diagnostic agents. Furthermore, these nanomedicines was often modified and extended by various means, such as improving the hypoxic environment in tumours by carrying oxygen.^79^ A recent study showed that hemoglobin (Hb) was linked to the photosensitizer chlorin e6 (Ce6) to construct a sorafenib (SRF, ferroptosis promoter)-loaded 2-in-1 nanoplatform (SRF@Hb-Ce6), which combined oxygen-promoted PDT and ferroptosis. More importantly, PDT enhanced ferroptosis by recruiting immune cells to secrete IFN-γ, which could sensitize tumours to ferroptosis.^80^*In vitro* results indicated that cell viability gradually decreases in the Ce6 (L) and Hb-Ce6 (L) groups with increasing Ce6 concentration, and there was a significant increase in cancer cell killing efficacy in the Hb-Ce6-treated group. In *in vivo* experiments, tumour-bearing mice treated with SRF@Hb-Ce6 had increased overall survival and significantly decreased tumour volume compared with other treatment groups. SRF@Hb-Ce6 created a platform for the collaboration of oxygen-enhanced PDT and ferroptosis treatment, which had co-promoting effects and exhibited high anti-tumour efficacy and safety for cancer treatment. The combination of the two therapies offered a promising strategy.

**Fig. 8:**
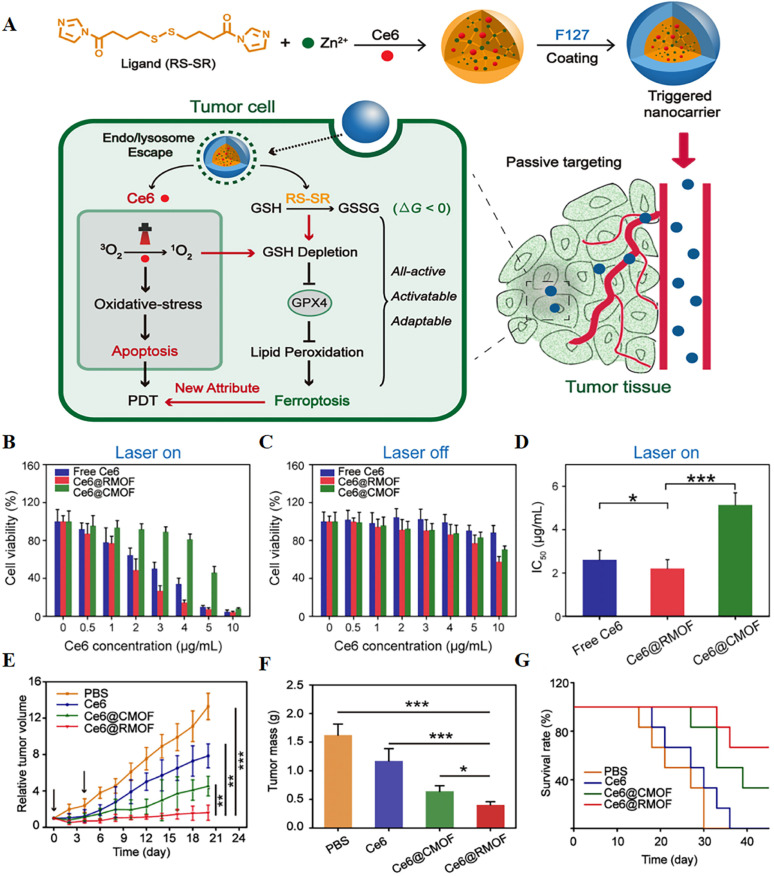
(A) Schematic illustration of a trigger-driven fully active metal–organic framework (MOF) carrier for combined apoptosis and ferroptosis anti-tumour photodynamic therapy (PDT). The disulfide of imidazole was coordinated with zinc ions to form an MOF, the photosensitizer Ce6 was successfully loaded, and the outer layer was wrapped with F127. When the nanocarriers reached the tumour site through passive targeting, singlet oxygen could be generated after near-infrared irradiation, thereby triggering apoptosis. At the same time, organic ligands could induce depletion of GSH, inactivation of GPX4, and ultimately ferroptosis. (B and C) Cytotoxicity of free Ce6, Ce6@CMOF and Ce6@RMOF with or without light irradiation in 4T1 cells. (D) Half-inhibitory concentrations of different treatment groups during laser treatment. (E) Tumour growth curves after treatment with PBS, free Ce6, Ce6@CMOF and Ce6@RMOF. (F) Quantitative analysis of tumour mass after 20 days of treatment. (G) Survival curves of mice treated with different treatment groups (*n* = 6). Reproduced with permission.^79^ Copyright 2019, American Chemical Society.

It is hypothesized that highly reactive singlet oxygen in PDT can deplete GSH and activate ferroptosis, and redox-responsive nanocarriers can further control this extent. To verify this, a fully active metal–organic framework (MOF) nanocarrier encapsulating a photosensitizer (Ce6) with a disulfide-bearing imidazole ligand coordinated with zinc was successfully prepared^79^ (Fig. 8). It was found that Ce6-loaded nanocarriers led to depletion of intracellular GSH *via* the disulfide–thiol exchange reaction in a mouse breast cancer cell line (4T1) regardless of light irradiation. GSH depletion further resulted in inactivation of GPX4 and enhanced cytotoxicity. *In vitro* experiments showed that free Ce6, Ce6@CMOF, and Ce6@RMOF were nontoxic at low doses in the absence of light irradiation. Fully active Ce6@RMOF exhibited some degree of toxicity at high doses due to ligand depletion by GSH. There was a significant difference in the half-maximal inhibitory concentration of Ce6@RMOF and Ce6@CMOF under laser-on conditions. The above results could be explained by the potency enhancement induced by GSH depletion and the rapid drug release of redox reactivity. Fully active nanocarriers exhibited superior anti-tumour activity *in vivo* due to cell apoptosis and ferroptosis.

#### 5.2.3 Lipid enriched nanomaterials

These nanomaterials can continuously trigger ferroptosis by enriching PUFAs in tumour cells/tumour micro-environments. This strategy of triggering ferroptosis is less noticeable, but very effective. A study by Ou *et al.* found that LDL-DHA nanoparticles were cytotoxic against both rat liver cancer and human hepatocellular carcinoma (HCC) cell lines.^81^ The results showed that the enhancement of lipid peroxidation and the reduction of GSH and the lipid antioxidant GPX4 in rat and human hepatoma cells after LDL-DHA treatment together resulted in cell death. Additionally, the effect of LDL-DHA treatment on human HCC tumour xenograft mice was also investigated. *In vivo* tumour inhibition experiments showed that intratumoural injection of LDL-DHA seriously inhibited the growth of HCC xenografts, which was consistent with the results of previous *in vitro* experiments. LDL-DHA induced HCC cell death through the ferroptosis pathway, which represented a novel molecular mechanism for the anticancer activity of LDL-DHA nanoparticles.

#### 5.2.4 Nanodrugs carrying non-coding RNA (ncRNA)

Recent studies have shown that genes can induce or inhibit ferroptosis through encoded proteins, and genetic technology can be used for ferroptosis-based cancer therapy. A large number of studies have reported the internal relationship between ncRNAs and ferroptosis, which provides a theoretical basis for our further experimental research. However, gene-interfering ferroptosis therapy may be a better measure through using ncRNAs to fight cancer than treatments that target ferroptosis using ncRNAs alone. Jiang *et al.*^8^ illustrated that p53 could mediate the regulatory process of ferroptosis in tumour cells, and proposed a new tumour suppressor model based on gene transfection (that is, p53 regulates cystine metabolism, ROS response and ferroptosis).

Yuan *et al.* demonstrated that ACSL4 is an essential ferroptosis gene, which could cause the death of ferritin cancer cells.^22^ In general, some genes (such as p53 and ACSL4) can become important factors or targets affecting ferroptosis by inhibiting or promoting the overexpression of some key proteins. Therefore, transfection of these genes into tumours can be used for ferroptosis treatment of cancer. The combined application of other nanomedicines and gene therapy shows certain advantages. A recent study well overcame tumour resistance to ferroptosis and achieved durable efficacy through the combination of RNA interference and ferroptosis-targeted nanomedicine.^82^ It shows that combining ncRNA-carrying nanomedicines with iron-based nanoparticles may provide good clinical results.

#### 5.2.5 Single atom catalysts

Recently, serial single atom catalysts have been proved to achieve ferroptosis. A single-atom Pd nanozyme for mild-temperature photothermal therapy of ferroptosis was reported by Chang *et al.*^83^ A nitrogen-coordinated carbon-supported Pd SAzyme was prepared by removing single atoms from metal nanoparticles. According to the principle of atomic economy, the Pd SAzyme exhibits mimetic activity and photothermal conversion properties similar to those of peroxidase (POD) and glutathione oxidase (GSHOx), which can lead to lipid peroxide (LPO) and ROS upregulated ferroptosis. More importantly, the production of large amounts of LPO and ROS inhibited the expression of heat shock proteins (HSPs), enabling Pd SAzyme-mediated mild PTT. *In vivo* anti-tumour results found that the Pd SAzyme + 1064 nm group exhibited significant tumour growth inhibition due to ferroptosis-promoted mild temperature PTT. Furthermore, the LPO and ROS generated during ferroptosis cleaved HSP70, and their expression was downregulated. More importantly, the SAzyme had good biosafety and no obvious toxicity, which provided a promising direction for future cancer treatment.

## 6. The effect of the tumour micro-environment on ferroptosis

The tumour micro-environment includes inflammatory response, tumour vasculature, stromal cells, immune cells, and metabolism,^84^ which are the important factors affecting the effect of the tumour treatment. A new strategy for treating tumours may involve targeting the tumour micro-environment to promote ferroptosis. A new study showed that CD8+ T cells in the tumour micro-environment not only induce cell death in the traditional way (perforin/granzyme pathway and Fas/Fas ligand-pathway), but also down-regulate SLC3A2 and SLC7A11 expression by secreting IFN to induce ferroptosis.^10^ Moreover, prostaglandin E2 (PGE2) has attracted widespread attention as an important immunomodulator. It has been shown that the induction of ferroptosis in cancer cells was associated with the increased expression of prostaglandin endoperoxide synthase 2 (PTGS2) and the release of PGE2,^19^ PGE2 and PTGS2 may promote ferroptosis in tumour cells. These studies revealed a new mechanism by which the immune system drove the ferroptosis pathway of cancer cells to inhibit tumourigenesis. In the future, the immune system can be used to promote the ferroptosis of tumour cells and develop relevant drugs to improve the effect of anti-tumour treatment.

## 7. Application of ferroptosis in cancer treatment

Different types of cancer cells have different susceptibilities to ferroptosis. Diffuse large B-cell lymphoma and renal cell carcinoma are more susceptible to erastin-induced ferroptosis than other cancer cells such as breast, lung, colon, melanoma, and ovarian cancer, according to the National Cancer Institute's Developmental Therapy Program.^17^ It has been suggested that the susceptibility of different cell lines to ferroptosis is different because of their different basal metabolic states. Numerous studies have confirmed the critical role of ferroptosis in killing cancer cells and inhibiting cancer growth. Further studies have shown that the combination of cytarabine, cisplatin, doxorubicin, temozolomide and other chemotherapeutic drugs with the ferroptosis inducer erastin had a significant synergistic effect on anti-tumour activity,^85^ and the prognosis was better than that of conventional chemotherapy alone.

Compared with normal cells, cancer cells have more vigorous metabolism, mitochondrial dysfunction, and more ROS accumulation, which increases the sensitivity of cancer cells to ferroptosis. In order to prevent ROS from causing damage to itself, cancer cells respond to stress and activate antioxidant mechanisms, such as superoxide dismutase (SOD), GPX4, and catalase, to remove excess ROS in cells.^86^ Therefore, induction of ferroptosis in cancer cells is of great significance for the treatment of cancer. On a global scale, ovarian cancer, colorectal cancer, diffuse large B-cell lymphoma, liver cancer, lung cancer, and other cancers frequently occur, seriously threatening the safety of human life. Although the mechanism of ferroptosis remains unclear, experiments have confirmed that ferroptosis could be activated through the TAZ-ANGPTL4-NOX2 signaling axis in ovarian cancer, thereby inhibiting the proliferation of ovarian cancer cells.^87^ Induction of ferroptosis by several potential anti-ovarian cancer small molecule drugs such as LY294002, sirolimus, and wortmannin may be a viable treatment option for ovarian cancer.^87^ Cytoglobin (CYGB) is a regulator of ROS and tumour suppressor that promotes lipid peroxidation on cell membranes, thereby inhibiting the growth of colorectal cancer cells. Colorectal cancer cells overexpressing CYGB are more sensitive to ferroptosis induced by RSL3 and erastin, and knockdown of the Yes-related protein 1 gene YAP1 can reduce the sensitivity of colorectal cancer cells to ferroptosis by reducing ROS production.^88^ Therefore, CYGB and YAP1 are regulatory nodes of ferroptosis sensitivity and provide potential targets for the treatment of colon cancer.

Among eight cell lines collected from various tissues, diffuse large B-cell lymphoma (DLBCL) cells were the most sensitive to ferroptosis-inducing agents.^19^ By stimulating necroptosis and ferroptosis, low doses of erastin could significantly increase chemotherapeutic agents' ability to kill non-APL acute myeloid leukemia (AML) cells. Non-APL AML cells were selectively sensitive to cytarabine and doxorubicin when exposed to erastin. Ferroptosis and necroptosis were induced synergistically in a mitogen-activated protein kinase (MAPK) dependent manner.^29^ Sorafenib can treat hepatocellular carcinoma (HCC) by inducing ferroptosis. Nrf2, is a key transcriptional regulator of ferroptosis, and its activity inhibition and expression *in vivo* and *in vitro* increased the anticancer activity of erastin and sorafenib in HCC cells. The p62-Keap1-Nrf2 pathway plays a crucial role in rescuing HCC cells from ferroptosis, and the Ras/Raf/MEK pathway has been reported to be a crucial target for ferroptosis treatment of HCC.^27^ It was found that knockdown of NQO1, HO1 and FTH1 (negative regulators of ferroptosis) could inhibit the growth of HCC cells following ferroptosis inducers. Cysteine desulfurase, an iron–sulfur cluster biosynthetic enzyme, could protect cells from ferroptosis under high oxygen tension by maintaining iron–sulfur cofactors.^89^ Furthermore, the deficiency of iron-sulfur clusters could activate the iron starvation reaction and combine with the inhibition of GSH biosynthesis to cause ferroptosis. *In vitro*, inhibiting NFS1 synergized with inhibition of cysteine transport to cause ferroptosis and slow tumour growth.

In addition, cancer treatment can be carried out by targeting amino acid, lipid, and iron metabolism-related signaling pathways. In amino acid metabolism, sulfasalazine can induce ferroptosis by inhibiting the X_c_^−^ system and reducing cystine uptake. p53 can regulate its expression by inhibiting the transcription of SLC7A11 *via* binding to the SLC7A11 promoter, thereby inducing ferroptosis, but its regulatory mechanism is still unclear.^90^ Studies have found that inhibiting the PI3K-AKT-mTOR signaling pathway could also induce ferroptosis in cancer cells.^91^ SLC7A11-mediated cystine uptake could also regulate ferroptosis by reducing GPX4 synthesis through the mTORC1 pathway.^92^

## 8. Conclusion and perspective

The currently discovered ferroptosis inducers include small molecules, natural compounds, nanomaterials, *etc.* Each of these drugs or methods has its own advantages and limitations.

Small molecules have received widespread attention because of their long shelf life and low toxicity. However, there are still some issues that deserve further consideration. The occurrence of ferroptosis may have adverse effects on normal tissues and cells due to the tumour-nonspecific association of small molecules. In addition, small molecules have short circulating half-lives in the blood and low effective drug concentrations at tumour sites due to the rapid renal clearance.^93^ Many natural compounds have been found to regulate ferroptosis, and natural compounds that promote ferroptosis are considered to have anti-tumour potential. Although natural compounds have the advantages of stable structure, high safety, and wide availability, they also have the disadvantages of different intensities of action and low specificity, which need to be weighed and evaluated by researchers.^12^ Since most of the current research on the induction of ferroptosis is still on a relatively shallow characterization, the exact regulatory targets and molecular mechanisms of natural compounds regulating ferroptosis and their structure–function relationships are still unclear. Therefore, in the future, on the one hand, it is urgent to confirm the occurrence of ferroptosis in multiple dimensions, and on the other hand, it is necessary to deeply explore the induction mechanism, and find the entry point at the gene or molecular level, so that the research results can be applied to the clinic and serve the clinic. Moreover, targeting the tumour micro-environment can also promote ferroptosis of tumour cells. However, because some tumour micro-environments such as inflammatory reaction has dual effects on cancer cell death, the intrinsic mechanism of the two opposite effects of inflammation related factors on tumour cell ferroptosis deserves further investigation. Furthermore, stromal cells, selenium metabolism and lactate metabolism exert inhibitory effects on the ferroptosis pathway.^94,95^ Therefore, it is a new method to search for anti-tumour drugs by exploring some factors that affect ferroptosis in the tumour micro-environment, studying its internal mechanism and combination with ferroptosis-inducing drugs. With further research on the influence of the tumour micro-environment on ferroptosis, the combination of ferroptosis-inducing drugs and drugs targeting the tumour micro-environment will open a new chapter of anti-tumour therapy.

In the past few decades, nanomedicines have made great progress in improving the solubility and stability of drugs, overcoming the limitations of pharmacokinetics, and reducing toxic side effects.^96^ Nanomaterials have the following advantages for cancer treatment of ferroptosis: (a) the precise targeting (passive and active targeting) of nanomaterials can reduce the adverse effects of ferroptosis on normal tissues and cells. As a result of the tumour vasculature's inherent features, namely enhanced permeability and retention (EPR) effects, passive targeting promotes the deposition of nanomaterials, which are not typically present in healthy tissues.^97^ Nanomaterials can also achieve the purpose of active targeting through the binding of antibodies or ligands.^98^ (b) Nanomaterials with a particle size greater than 6 nm can reduce renal clearance and prolong blood flow. It can effectively prolong the half-life of circulation and enhance the effective accumulation at the tumour site. However, due to the possibility of unstable and easy to form aggregates, the shelf life of nanomaterials is relatively short. In addition, the residual non degradable nanomaterials in the body may also have long-term toxicity risk. While existing nanomaterials have produced good ferroptosis effects there are still many challenges to overcome before they can be used clinically. First, new ferroptosis-inducing nanomaterials should be continuously developed to find optimal materials. Secondly, iron loading and release should be increased to promote iron levels in cancer cells. Lastly, to ensure biosafety, nanomaterials should be made biocompatible, biodegradable, and immunogenic. In general, although ferroptosis-induced nanomaterials can achieve ideal tumour inhibition, almost all research data are derived from experimental animals, so there is still much work to be done.

A large number of studies have shown that ferroptosis can kill cancer cells and inhibit the growth of tumour cells. The discovery of ferroptosis provides a new idea for cancer treatment and drug resistance. Although research on ferroptosis continues to increase, some questions remain. For example, ferroptosis has certain characteristics of tumour heterogeneity and gene selection, and its occurrence mechanism involves the expression regulation of multiple genes and the interaction of different signaling pathways. Therefore, it is of great significance to further study the regulatory effect and molecular mechanism of different genes on ferroptosis in various cancer diseases, and to develop new anti-cancer drugs based on ferroptosis in a targeted manner. Despite the rapid development in the field of ferroptosis-based cancer therapy, the potential clinical application of ferroptosis-based nanotherapeutics remains challenging due to the following issues. First, different cancer cell lines have different susceptibilities to ferroptosis; there may be a large gap between animals and humans in this non-apoptotic mode of cell death, and the choice of experimental model must be carefully considered. It should also be remembered that ferroptosis can be used not only in cancer treatment but it may also cause cancer and other diseases. Second, potential toxic side effects of ferroptosis inducers or enhancers should be fully investigated to ensure tumour-specific triggering of the Fenton response and circumvent off-target toxicity to normal tissues. In addition, ferroptosis is a process regulated by cellular metabolism, and minor changes in key molecules and metabolites that regulate ferroptosis, such as iron, GSH, PUFAs, *etc.*, will affect the sensitivity of target cells to ferroptosis. Finally, the rational design of ferroptosis-based combination nanotherapeutics may have important implications for their synergistic effects, possible additive toxicity, and industrial viability of complex nanostructures. Therefore, multiple issues should be considered in the rational design of effective and safe nanotherapeutics based on ferroptosis in the future.

The rational design of efficient and safe anti-tumour drugs based on ferroptosis in the future should consider many aspects. Research and clinical translation of ferroptosis are still largely unknown and challenging. With the deepening of basic research on ferroptosis, we believe that it will be possible to apply ferroptosis inducers or enhancers to the clinic to sensitize tumour therapy. Nanomaterials in particular serve as an excellent platform that can be carefully engineered and integrated into a microcarrier for bioimaging and drug delivery. Although ferroptosis-mediated cancer treatment research is still in its infancy, it is receiving increasing attention, and interdisciplinary collaboration is expected to promote the development of ferroptosis-related research. Future research results around the induction of ferroptosis will surely bring good news to patients with tumours and other diseases.

## Author contributions

Each named author has substantially contributed to drafting and revising this manuscript.

## Conflicts of interest

The authors declare that they have no known competing financial interests or personal relationships that could have appeared to influence the work reported in this paper.

## Supplementary Material
